# LncNFYB promotes the proliferation of rheumatoid arthritis fibroblast-like synoviocytes *via* LncNFYB/ANXA2/ERK1/2 axis

**DOI:** 10.1016/j.jbc.2023.105591

**Published:** 2023-12-21

**Authors:** Shibai Xiao, Qingqing Ouyang, Yi Feng, Xiaoxi Lu, Yipeng Han, Hao Ren, Qin Huang, Jinjun Zhao, Changhong Xiao, Min Yang

**Affiliations:** 1 Nanfang Hospital, Southern Medical University, Guangzhou, China; 2 Department of Rheumatology and Immunology, Integrated Hospital of Traditional Chinese Medicine, Southern Medical University, Guangzhou, China

**Keywords:** rheumatoid arthritis, fibroblast-like synoviocytes, lncRNA, ANXA2, ERK1/2

## Abstract

Long noncoding RNAs (lncRNAs) are specifically expressed in different diseases and regulate disease progression. To explore the functions of rheumatoid arthritis (RA)-specific lncRNA, we determined the lncRNA expression profile of fibroblast-like synoviocytes (FLS) obtained from patients with RA and osteoarthritis (OA) using a LncRNA microarray and identified up-regulated LncNFYB in RA as a potential therapeutic target. Using gain- and loss-of-function studies, LncNFYB was proven to promote FLS proliferation and cell cycle progress but not affect their invasion, migration, and apoptotic abilities. Further investigation discovered that LncRNA could combine with annexin A2 (ANXA2) and enhance the level of phospho-ANXA2 (Tyr24) in the plasma membrane area, which induced the activation of ERK1/2 to promote proliferation. These findings provide new insights into the biological functions of LncNFYB on modification of FLS, which may be exploited for the therapy of RA.

Rheumatoid Arthritis (RA) is a chronic autoimmune disease that affects 1% of the global population (1). This inflammatory disease mainly manifests as erosive and symmetrical polyarthritis, and exhibits several pathological features including synovial tissue hyperplasia, inflammatory cell infiltration, angiogenesis, pannus formation, and progressive destruction of the articular cartilage and bone (2). The pathogenesis of RA is complex and has not yet been fully elucidated.

Fibroblast-like synoviocytes (FLS) play a vital role in inflammatory reactions and joint injury associated with the pathogenesis of RA (3). Joints are covered with a synovial membrane, which is normally thin and comprised of two or three layers of FLS (4). In RA, this lining is transformed into a vascular structure, with a large number of activated FLS, which promotes joint destruction because of high malignancy associated with enhanced migration, invasion, and proliferation of FLS (5). As such, an understanding of the mechanisms underlying FLS proliferation in RA may help identify potential therapeutic targets.

Long noncoding RNAs (lncRNA) are a group of RNAs that are not translated into proteins but can modify the activities of cells in conjunction with RNA-binding proteins (RBPs) or microRNAs (6). The expression of lncRNAs shows cell, tissue, and disease specificity, which renders them as a biomarker of the type and stage of diseases and cells (7, 8). LncRNA *XIST* transcribed from the X-inactive specific transcript (XIST) gene is a well-known lncRNA that is regarded as a diagnostic and prognostic biomarker for a variety of tumor types, including leukemia, lung cancer, breast cancer, and liver cancer (9). Differentially expressed lncRNAs may regulate the occurrence and development of diseases (10). LncRNAs have been reported to regulate migration, invasion, proliferation, apoptosis, and immune responses of FLS in RA (11). The ability of lncRNAs to promote or prevent the disease progression makes them potential therapeutic targets.

Annexin A2 (ANXA2) is a member of the annexin family, which is expressed in almost all cells and exhibits various functions, including those related to cell invasion and metastasis, bleeding disorders, angiogenesis, and induction of inflammatory factors (12). However, its role and mechanism of action in RA-FLS were not well understood. As an RBP, the effects of ANXA2 are regulated by lncRNAs. For example, ANXA2 promotes apoptosis in combination with *lnc- Fendrr* (13). Also, it binds to LINC00941 and abnormally activates the FAK/AKT pathway (14).

In this study, we explored the involvement of lncRNAs in RA pathogenesis and tried to decipher the mechanism underlying their function. We identified highly expressed lncRNAs of RA FLS, LncNFYB, which could interact with ANXA2 and increase its phosphorylation. P-ANXA2 activates downstream extracellular signal-regulated protein kinase half (ERK1/2), which contributes to cell proliferation. We propose the LncNFYB/ANXA2/ERK1/2 axis as a theoretical foundation for potential therapeutic targeting of RA.

## Results

### Identification of lncRNAs in RA FLS

**Figure 1:**
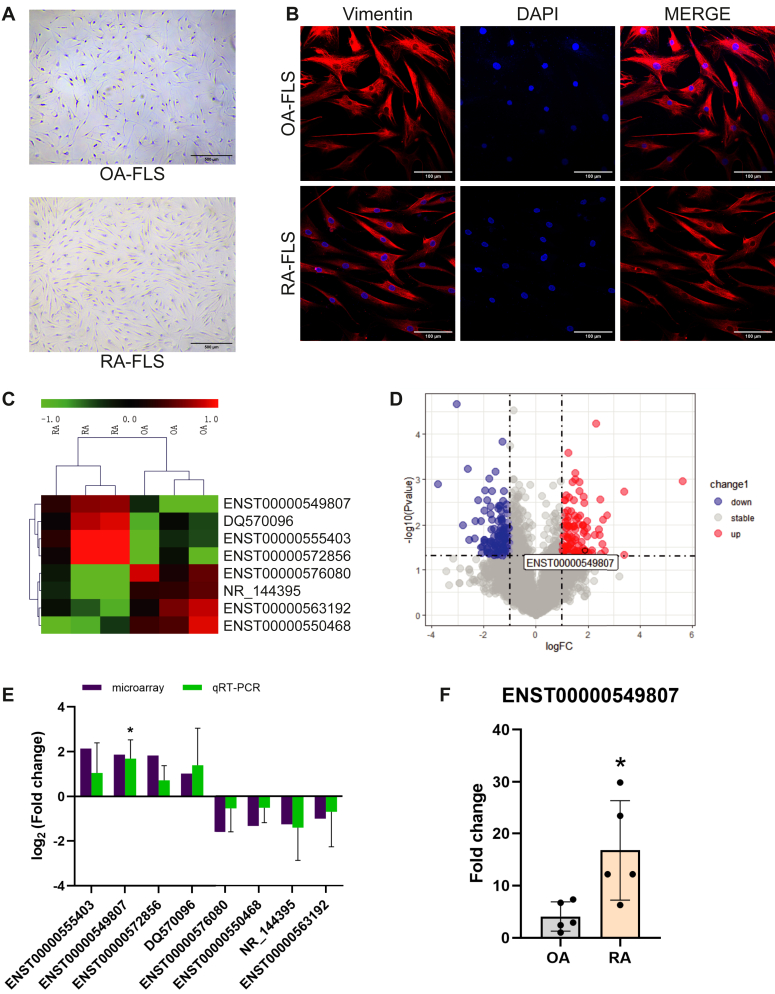
**LncRNA microarray of OA and RA FLS.***A*, morphology of RA-FLS and OA-FLS under light microscope. *B*, RA-FLS and OA-FLS vimentin immunofluorescence staining. *Red* shows vimentin staining; blue shows the nuclear staining. *C*, in the cluster diagram of the lncRNA microarray, the first three columns show lncRNA expression in RA-FLS from three patients, and the last three columns show lncRNA expression in OA-FLS from three patients. *Green* and *red* indicate relatively *low* and *high* expression levels, respectively. *D*, the volcano plot for the lncRNA microarray; >2.0-fold change and *P* -value <0.05 were criteria for differential expression. Green and red represent lncRNAs with low and high expressions in RA-FLS compared with OA-FLS. *E*, the fold changes of *ENST00000555403*, *ENST0000054980*, *ENST000005728*, *DQ570096*, *ENST0000057608*, *ENST0000055046*, *NR_14439*, and *ENST00000563192* in RA and OA FLS were chosen to validate the LncRNA microarray data by qRT-PCR. The *black bars* represent microarray data. And the *green bars* represent mean values of log_2_-fold change from qRT-PCR data. *F*, the expression of ENST0000054980 in RA and OA FLS from five patients obtained from qRT-PCR. ∗ indicates *p* < 0.05 compared with the control group. Data are represented as mean ± SEM (n = 5).

FLS were isolated from patients with RA and OA who satisfied the 2010 RA and OA diagnostic criteria, respectively. The cultured cells were length about 200 μm, appeared triangular, polygonal, or fusiform, with a central oval nucleus, showing an appearance of fibrocytes. Besides, RA-FLS had more cytoplasmic content and compact arrangement than that OA-FLS (Fig. 1*A*). Both RA and OA FLS cultured *in vitro* were identified based on immunofluorescence detection of vimentin, which is characteristically expressed at high levels in fibroblasts. Strong signal of vimentin located in the whole cytoplasm, illustrated the cells were FLS (Fig. 1*B*).

To identify the lncRNA expression profile of RA-FLS, we performed a microarray analysis of RA and OA FLS (3 samples for RA-FLS, 3 samples for OA-FLS) (Supplementary Figure*A*). The volcano plot shows 159 upregulated and 304 downregulated lncRNAs in RA-FLS compared with that in OA FLS, with a >2.0-fold change and *p*-value <0.05 (Fig. 1*D*). In the cluster diagram of the lncRNA microarray, green and red indicate relatively low and high expression levels, respectively. Based on greater specific fold-change in expression, four upregulated lncRNAs, namely *ENST00000555403*, *ENST00000549807*, ENST0000057285, and DQ570096, and four downregulated lncRNAs, *ENST00000576080*, *ENST00000550468*, *NR_144395*, and *ENST00000563192*, were chosen as candidate biomarkers or therapeutic targets for further investigations (Fig. 1*C*).

We evaluated the expression levels of the chosen lncRNAs in FLS obtained from five patients each with RA and FLS using qRT-PC (Fig. 1*E* and Supplementary Figure*B*). The expression of *ENST00000555403*, *ENST00000549807*, ENST0000057285, and DQ570096 in RA-FLS was 4.41-, 3.651-, 3.549 and 2.03-times higher than in OA-FLS in the microarray analysis and 2.533-, 4.116-, 1.089 and 2.483-times higher as determined using qRT-PCR. With regard the downregulated lncRNAs, the expression level of *ENST00000576080*, *ENST00000550468*, *NR_144395*, and *ENST00000563192* in OA-FLS was 3.008-, 2.501-, 2.382-, and 1.991-times higher than in RA-FLS as determined using the microarray analysis, and 1.631-, 1.412-, 4.016-, and 2.899-times higher in qRT-PCR analysis.

For further experiments, we focused on lncRNA *ENST00000549807* (Fig. 1*F*), the expression of which in RA-FLS was 4.116-times higher than in OA FLS, consistent with the 3.65-times higher expression in the microarray results. It was the most diverse expression one among these candidate lncRNAs.

### Basic information about *ENST00000549807*

**Figure 2:**
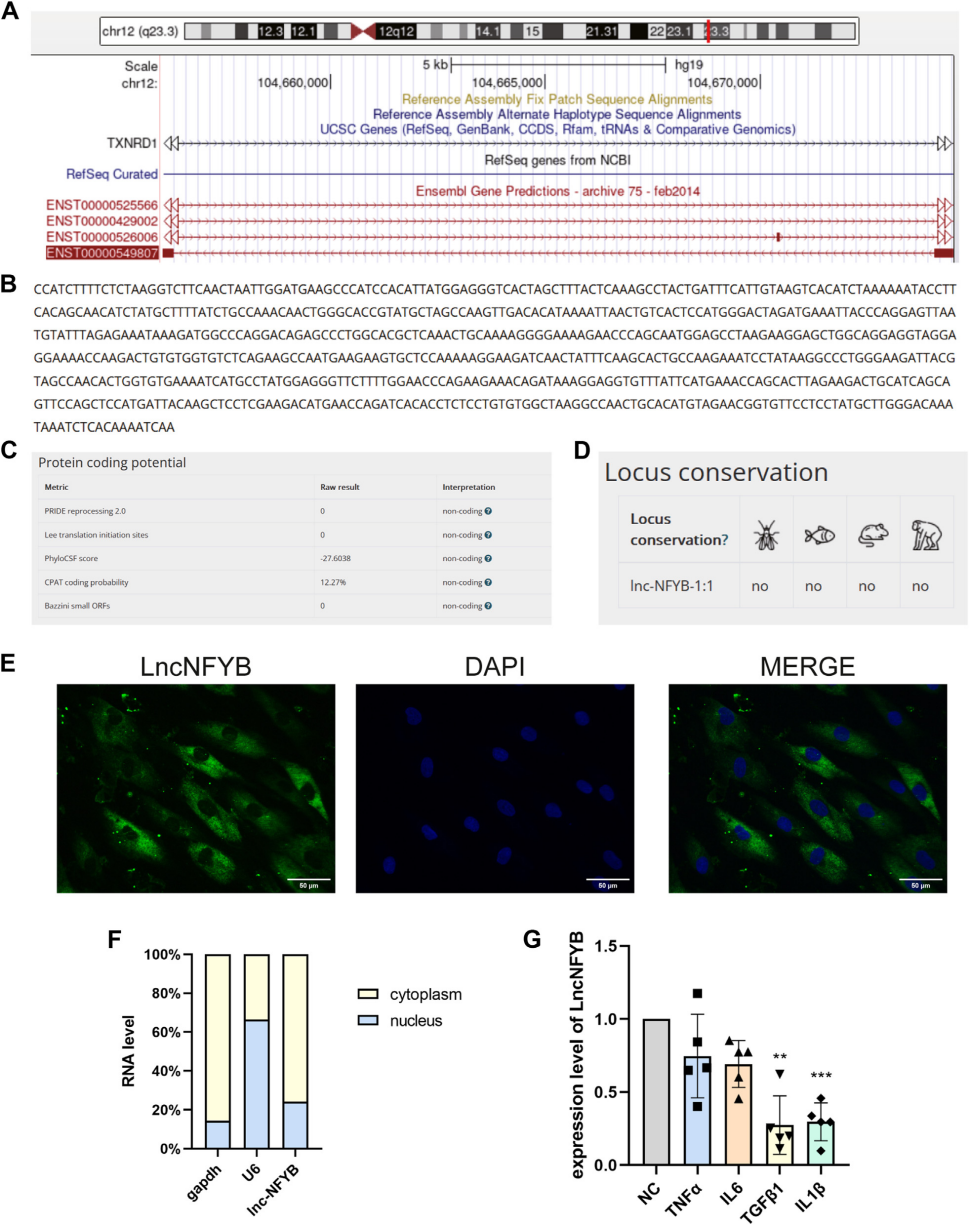
**Basic information about LncNFYB.***A*–*D*, the origin genes (*A*), full-length sequence (*B*), prediction of coding capability (*C*), and conservation analysis (*D*) for LncNFYB. *E*, FISH for LncNFYB, *green* fluorescence shows LncNFYB expression and blue fluorescence shows nuclear staining. *F*, expression of LncNFYB tested by qRT-PCR after nuclear/cytoplasmic fractionation. *G*, expression of LncNFYB of RA-FLS after TNFα, IL-6, TGFβ1, and IL 1β stimulation. ∗∗ and ∗∗∗ indicate *p* < 0.01, and 0.001, respectively, compared with the control group. Data are represented as mean ± SEM (n = 5).

As described in the NCBI database (http://genome.ucsc.edu/), *ENST00000549807* is a 701 bp lncRNA transcribed from chr12(q23.3) (Fig. 2, *A* and *B*). In the LNCipedia database (https://lncipedia.org/), it is also referred to as LncNFYB, which we use hereafter. Various software analyses revealed that LncNFYB cannot be transcribed into a protein and has no homologous sequence in *Drosophila*, zebrafish, rat, and monkey (Fig. 2, *C* and *D*). In the FISH assay, a high-intensity signal for LncNFYB was observed in the cytoplasmic region (Fig. 2*E*). Consistent with this result, we detected 75.7% of the total LncNFYB in the cytoplasm and 24.3% in the nucleus using qRT-PCR after nuclear-cytoplasmic fractionation of RNA (Fig. 2*F*).

OA and RA joints are characterized by chronic inflammation, and the level of inflammation changes constantly in response to several factors besides the disease itself. To rule out the difference in LncNFYB expression caused by different levels of inflammatory stimulation in the affected joint, we detected LncNFYB levels in RA-FLS after stimulation with various pro-inflammatory factors. As far as known until the present, the TNFα and IL-6 are the most important pro-inflammatory factors in RA. The expression of LncNFYB was not altered by TNFα and IL-6 but was decreased by TGFβ1 and IL 1β, which indicates that the increased LncNFYB levels in RA-FLS are not because of the different levels of inflammatory stimulation but most likely due to the activation of RA-FLS (Fig. 2*G*).

### LncNFYB promoted the proliferation of RA and OA FLS

To explore the role of LncNFYB in RA-FLS, we designed two different siRNAs to knock down its expression (Supplementary Figure*C*). si-LncNFYB-1 and si-LncNFYB-2 could knock down the expression of LncNFYB by 90.4% and 73.7%, respectively. In addition, we used an adenovirus to overexpress LncNFYB, which induced a hundred-fold increase at a MOI of 20 (Supplementary Figure*D*). The knockdown and overexpression of LncNFYB in RA-FLS were used to explore its functions in proliferation, migration, invasion, and apoptosis.

**Figure 3:**
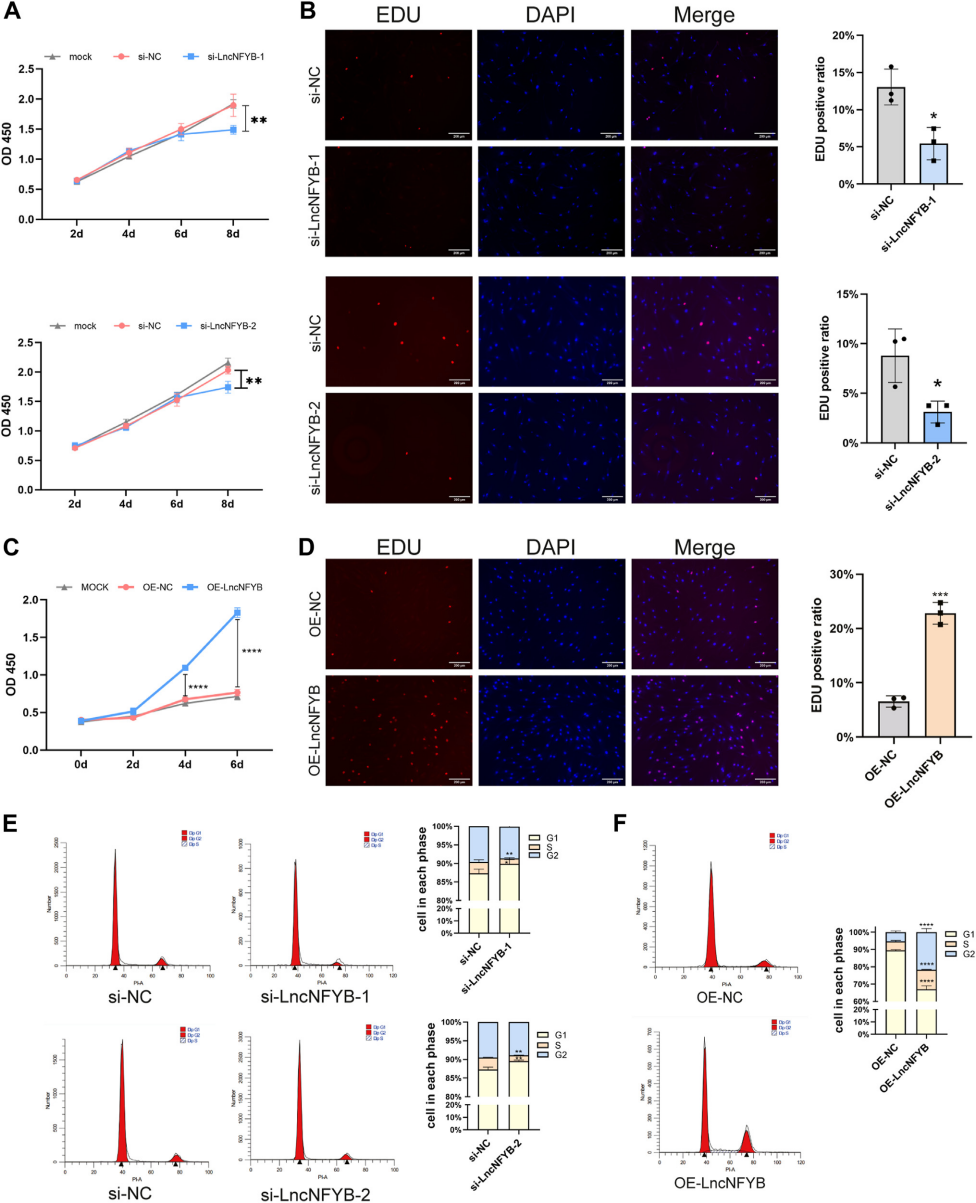
**LncNFYB promoted the proliferation of RA FLS.***A* and *B*, quantification of CCK-8 proliferation assays (*A*) and EDU staining (*B*) of RA FLS following LncNFYB knockdown. *C* and *D*, quantification of CCK-8 proliferation assays (*C*) and EDU staining (*D*) of RA FLS following LncNFYB overexpressing. *E* and *F*, cell cycle distribution of RA-FLS after LncNFYB knocking down (*E*) or overexpressing (*F*). "OE" indicates over-expression. ∗, ∗∗, ∗∗∗, and ∗∗∗∗ indicates to *p* < 0.05, 0.01, 0.001, and 0.0001 compared with the control group. Data are represented as mean ± SEM (n = 3).

The proliferation ability of FLS was determined using the CCK8 assay and was found to decrease in LncNFYB-deficient conditions for 8 days (Fig. 3*A*). Furthermore, the EdU assay showed fewer cells with EdU-positive signals after LncNFYB knockdown, indicating fewer cells in the dividing phase (Fig. 3*B*). Flow cytometry analysis showed that more cells remained in the G1 phase after LncNFYB knockdown than in the negative control group (Fig. 3*E*). These results strongly suggest that the knockdown of LncNFYB inhibits the proliferation of RA-FLS.

We also overexpressed LncNFYB in RA-FLS *via* adenoviral infection. The same set of experiments including CCK8 assay, EdU assay, and cell cycle analysis, was carried out on these LncNFYB-overexpressing cells. Compared with the empty vector control group, 4 days after LncNFYB overexpression, the OD450 value increased significantly and progressively over time indicating increased proliferation (Fig. 3*C*). The EdU-positivity rate in the control group was 6.59%, which was almost three times higher (up to 22.78%) than that in the experimental group (Fig. 3*D*). Flow cytometry analysis revealed that less RA-FLS were in the mitotic phase than that in the LncNFYB overexpressing group (Fig. 3*F*). All these results indicate the proliferation-promoting ability of LncNFYB.

**Figure 4:**
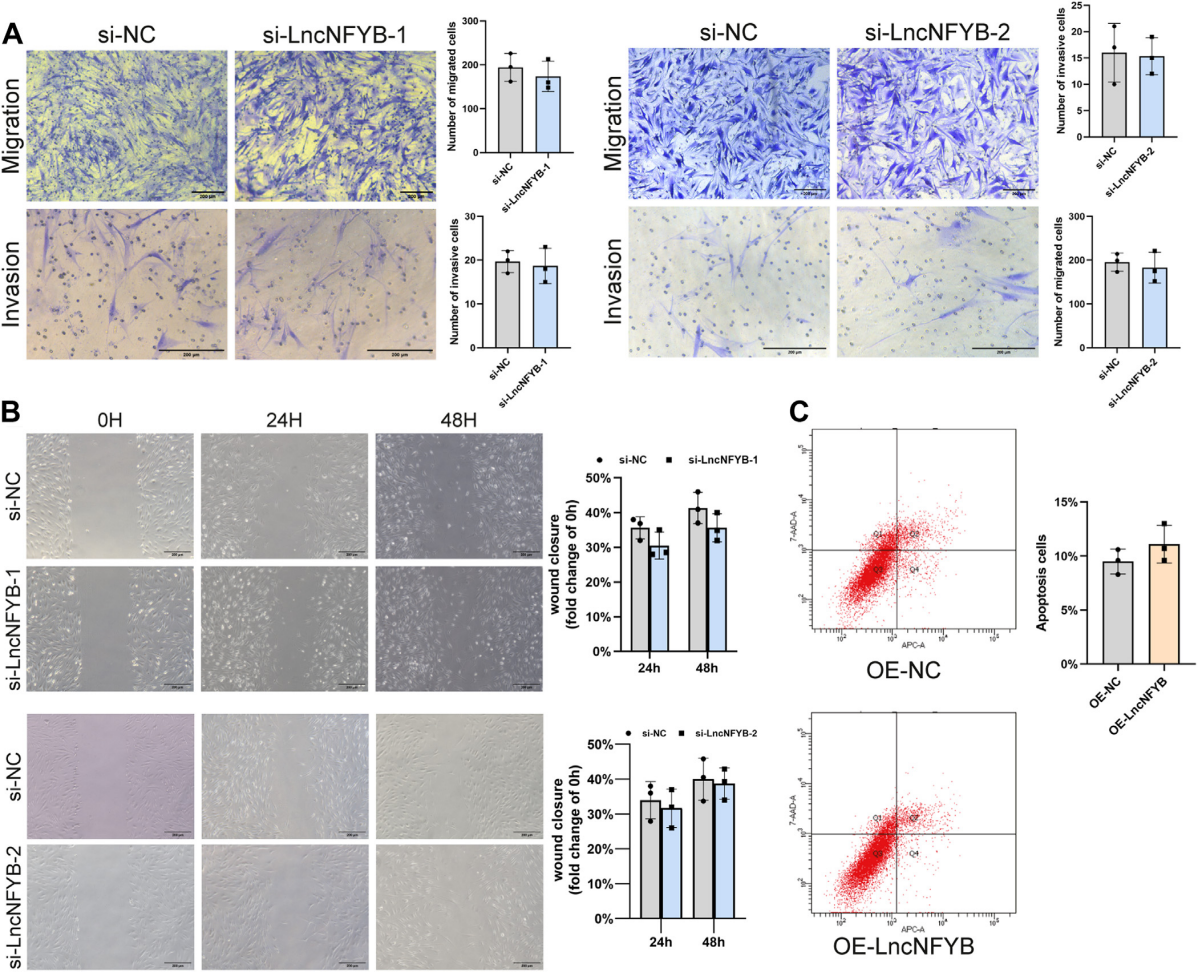
**LncNFYB did not affect the migration, invasion, and apoptosis of RA-FLS.***A*, transwell migration and invasion were carried after si-LncNFYB treatment. *B*, wound-healing experiment were conducted after LncNFYB was knocked down. *C*, flow cytometry tested the apoptosis of RA-FLS after overexpressing LncNFYB. “OE” indicates over-expression. Data are represented as mean ± SEM (n = 5).

The effect of LncNFYB on the migration, invasion, and apoptotic abilities of RA-FLS was also evaluated. For LncNFYB-deficient RA-FLS, both transwell assay and scratch test showed no changes in migration and invasion ability (Fig. 4, *A* and *B*). Meanwhile, the proportion of apoptotic cells was unchanged after LncNFYB overexpression (Fig. 4*C*). These results indicated that LncNFYB did not affect the migration, invasion, and apoptotic capacity of RA-FLS.

**Figure 5:**
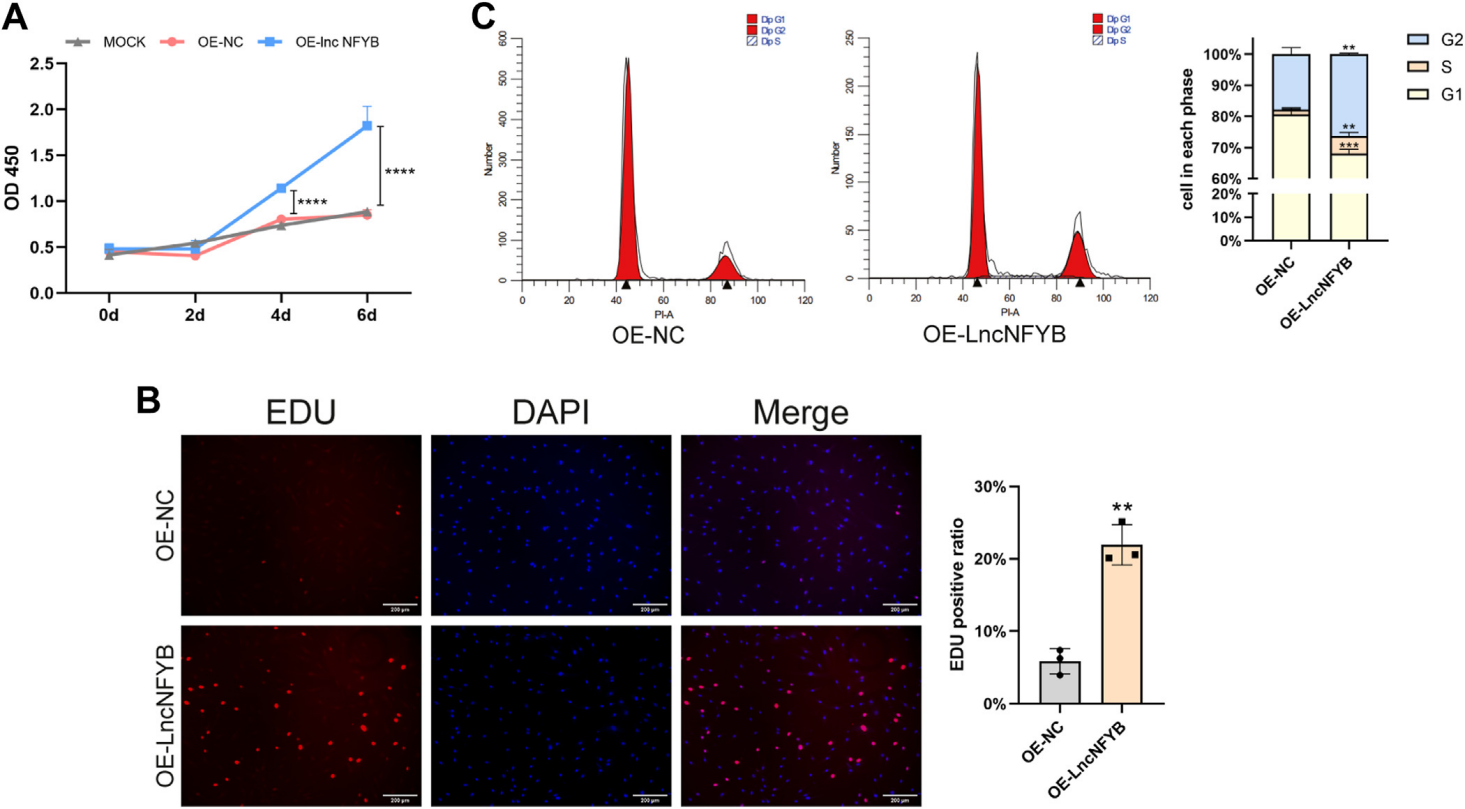
**LncNFYB promoted the proliferation of OA FLS.***A–C*, quantification of CCK-8 proliferation assays (*A*), EDU staining (*B*) and cell cycle distribution (*C*) of OA FLS following LncNFYB overexpressing "OE" indicates over-expression. ∗, ∗∗, ∗∗∗, and ∗∗∗∗ indicates to *p* < 0.05, 0.01, 0.001, and 0.0001 compared with the control group. Data are represented as mean ± SEM (n = 3).

Compared with RA-FLS, OA-FLS has a weaker proliferative capacity, and the downregulating LncNFYB might be a cause. To test this hypothesis, we investigated the proliferation level of OA-FLS with overexpressing LncNFYB. Likewise，in OA-FLS, the increased LncNFYB caused enhanced proliferation ability. Ectopic LncNFYB in OA-FLS increased the absorbance in the CCK8 assay, the positivity rate in the EDU assay, and the proportion of cells in the mitotic phase (Fig. 5, *A–C*).

Overall, these results show that LncNFYB promoted the proliferation of FLS in RA but did not affect the migration, invasion, or apoptosis of these cells.

### LncNFYB could bind to ANXA2

**Figure 6:**
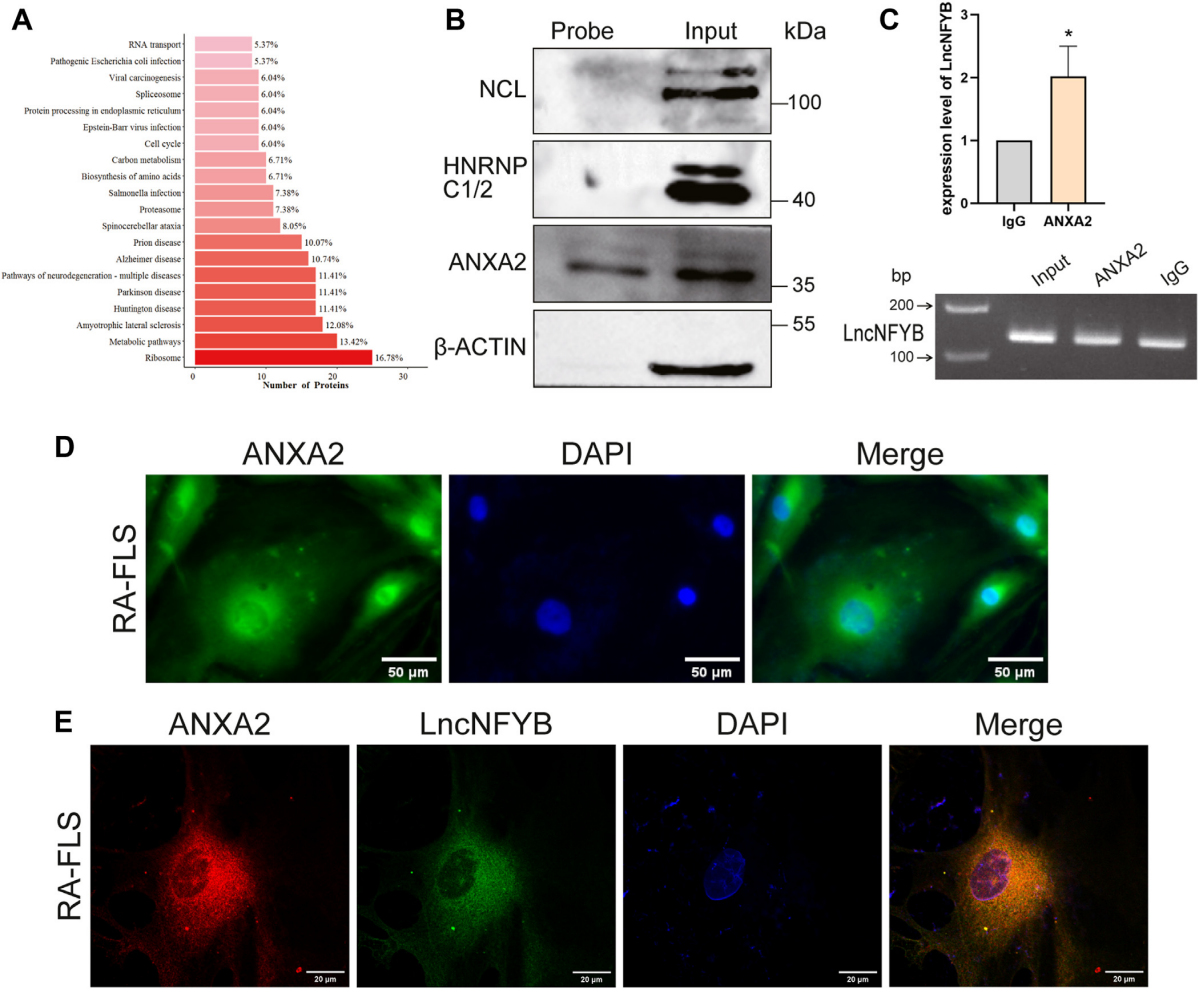
**LncNFYB bound to ANXA2.***A*, the KEGG pathway of LncNFY pulled-down proteins were analyzed. *B*, western blotting results for LncNFYB pull-down proteins, HNRNPC1/2, NCL, and ANXA2. *C*, Amount of LncNFYB pulled down in the ANXA2-RIP experiment and the agarose electrophoresis of pull-down product after LncNFYB specific qRT-PCR amplification. *D*, ANXA2 immunofluorescence staining in RA-FLS. *E*, fluorescence assessment of ANXA2 and LncNFYB co-localization in RA-FLS. ∗ indicates *p* < 0.05 compared with the control group. Data are represented as mean ± SEM (n = 3).

LncRNAs are known to interact with RBPs to regulate cellular activities. To explore whether LncNFYB promotes RA-FLS proliferation through this mechanism, we performed RNA pull-down and mass spectrometry. In the KEGG enrichment analysis of the pulled-down proteins, “ribosome” accounted for the largest proportion (Fig. 6*A*). Notably, 6.04% of the terms were associated with cell cycle. The GO analysis and the protein-protein interaction network of proliferation-associated proteins from LncNFYB pulled-down were shown in the supplementary figure (Supplementary Figure, *E* and *F*).

Among the pulled-down proteins, we chose three candidate RBPs associated with LncNFYB, namely HNRNP C1/2, NCL, and ANXA2. Interestingly, ANXA2 was the only protein that bound with LncNFYB that could be detected using Western blotting (Fig. 6*D*). The amount of LncNFYB precipitated with the ANXA2 antibody was higher than that precipitated with lgG in RIP, which further confirmed the combination of ANXA2 and LncNFYB (Fig. 6*E*).

ANXA2 belongs to the annexin family and is widely expressed in various cells. It is mainly localized in the cell membrane and cytoplasm but is also present in the nucleus. In RA-FLS, ANXA2 was distributed throughout the cell, including the nucleus, perinuclear region, and plasma membrane, among which the perinuclear region has the most distribution (Fig. 6*F*). Immunofluorescence analysis of ANXA2 and LncNFYB showed their co-localization in RA-FLS, proved the combination again (Fig. 6*G*).

### LncNFYB promoted the phosphorylation of ANXA2 on Tyr24

**Figure 7:**
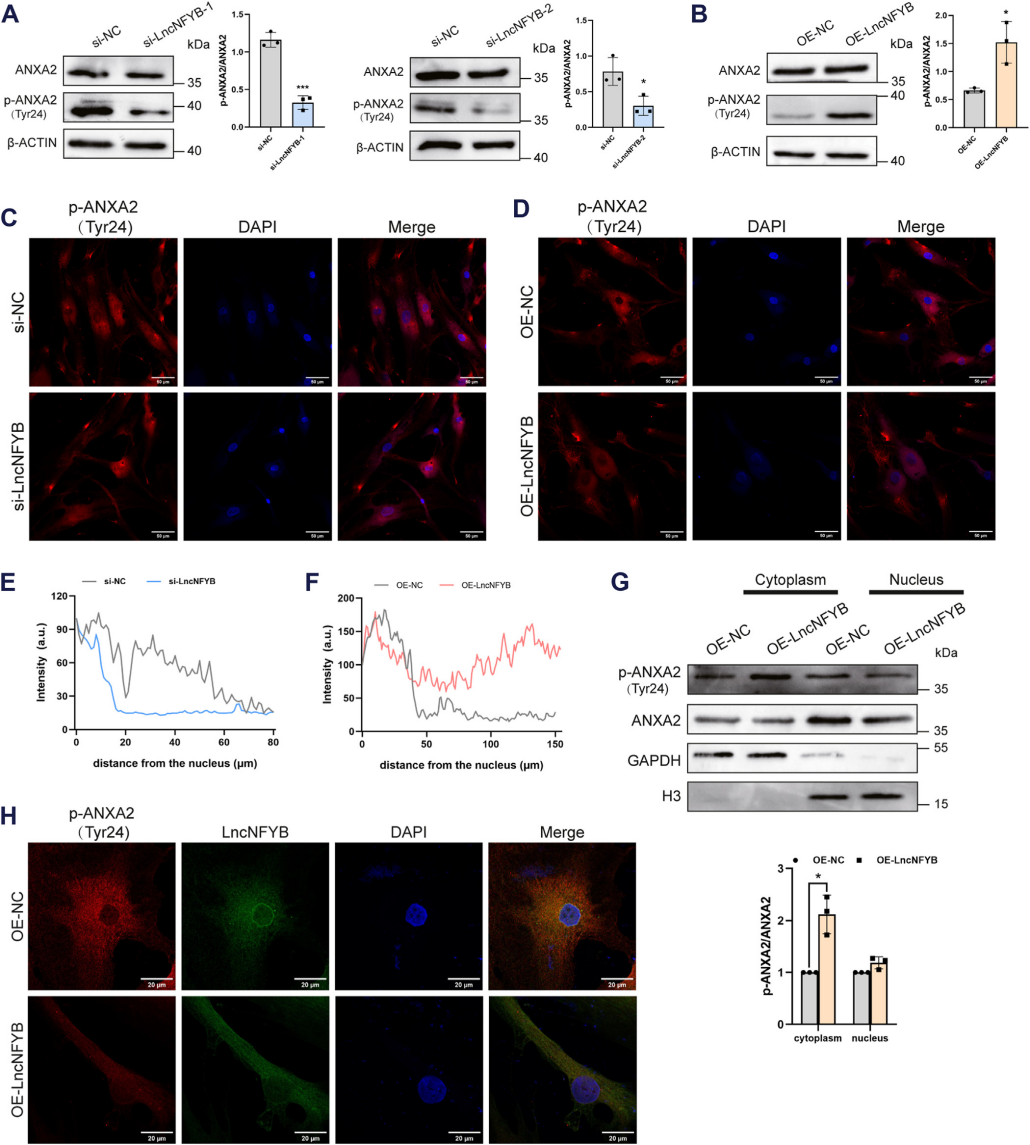
**LncNFYB promoted the phosphorylation of ANXA2(Tyr24) in plasma membrane region.***A* and *B*, the protein expression of total ANXA2 and p-ANXA2 (Tyr24) after LncNFYB knockdown (*A*) and overexpression (*B*). *C* and *D*, immunofluorescence staining (*C*) and intensity distribution (*D*) of p-ANXA2 (Tyr24) after LncNFYB knockdown. *E* and *F*, immunofluorescence staining (*E*) and intensity distribution (*F*) of p-ANXA2 (Tyr24) after LncNFYB overexpression. *G*, expression of ANXA2 and p-ANXA2 (Tyr24) in cytoplasm and nucleus after LncNFYB overexpression. *H*, fluorescence assessment of p-ANXA2 (Tyr24) and LncNFYB co-localization in LncNFYB overexpressed RA-FLS. "OE" indicates over-expression. ∗ and ∗∗∗ indicate *p* < 0.05 and 0.001, respectively, compared with the control group. Data are represented as mean ± SEM (n = 3).

To investigate how LncNFYB affects ANXA2, we compared the expression levels of ANXA2 in both LncNFYB-knockdown and -overexpressing groups of RA-FLS. The ANXA2 mRNA levels did not change in either of the groups (Supplementary Figure, *E* and *F*). We then tested the total protein expression of ANXA2, which was unaffected by the knockdown or overexpression of LncNFYB (Fig. 7, *A* and *B*). However, the level of phospho-ANXA2 (p-ANXA2) at Tyr24 was reduced in LncNFYB-deficient RA-FLS and enhanced in LncNFYB-overexpressing cells (Fig. 7, *A* and *B*).

Ser25 and Tyr24 are the most common phosphorylation sites in ANXA2. According to a previous study, p-ANXA2 (Tyr 24) is abundant in the plasma membrane and nuclear region, whereas p-ANXA2 (Ser 25) is abundant in the perinuclear region (15). In breast cancer, increased levels of p-ANXA2 (Tyr 24) in the cytoplasm promote cancer cell proliferation (16, 17). In our study, p-ANXA2 (Tyr 24) was mainly distributed within 20 μm of the nucleus in the si-LncNFYB treatment group and within 60 μm in the control group (Fig. 7, *C* and *D*). Besides, content of p-ANXA2 (Tyr 24) increased in areas 100 to 150 μm away from the nucleus in OE-LncNFYB group than that in the control group (Fig. 7, *E* and *F*). The result that p-ANXA2 (Tyr 24) located more intensively in the perinuclear region after LncNFYB knockdown, and more dispersed to the plasma membrane region after LncNFYB overexpression, was consistent with a previous study showing that p-ANXA2 (Tyr 24) often occurs in the plasma membrane region. Proteins isolation showed that LncNFYB only promoted the phosphorylation of ANXA2 in the cytoplasm, which was consistent with the cytoplasmic location of LncNFYB (Fig. 7*G*). Double immunofluorescent staining revealed the co-localization of p-ANXA2 (Tyr 24) and LncNFYB in LncNFYB overexpressing group and the control group (Fig. 7*H*). These results indicated the LncNFYB combination of ANXA2 promoted the plasma membrane position of p-ANXA2 (Tyr 24).

### LncNFYB promoted the activation of ERK1/2 in RA-FLS *via* phosphorylated ANXA2

According to previous studies, in the plasma membrane region, p-ANXA2 (Tyr 24) could interact with membrane proteins, facilitating the transduction of extracellular signals inside the cells (18, 19). In fibroblasts and smooth muscle cells, increased p-ANXA2 (Tyr 24) levels promoted the phosphorylation of ERK1/2 (p-ERK1/2), which further facilitated proliferation, migration, and invasion of cells (20). To test whether this also occurred in RA-FLS, we designed an overexpression and two mutant plasmids of ANXA2, ANXA2-WT (wild type), ANXA2-Y24A (phospho-refractory mutant), and ANXA2-Y24D (phosphorylation-mimicking mutant). These plasmids were transfected into RA-FLS and total RNA and protein were isolated after 48 h. The results of qRT-PCR analysis showed that the levels of ANXA2 mRNA in RA-FLS overexpressing ANXA2-WT, ANXA2-Y24A, and ANXA2-Y24D were higher than that in the control group (Figure supplement*G*). Besides, Western blot analysis showed the exogenous ANXA2 was expressed in RA-FLS (Figure supplement*H*).

**Figure 8:**
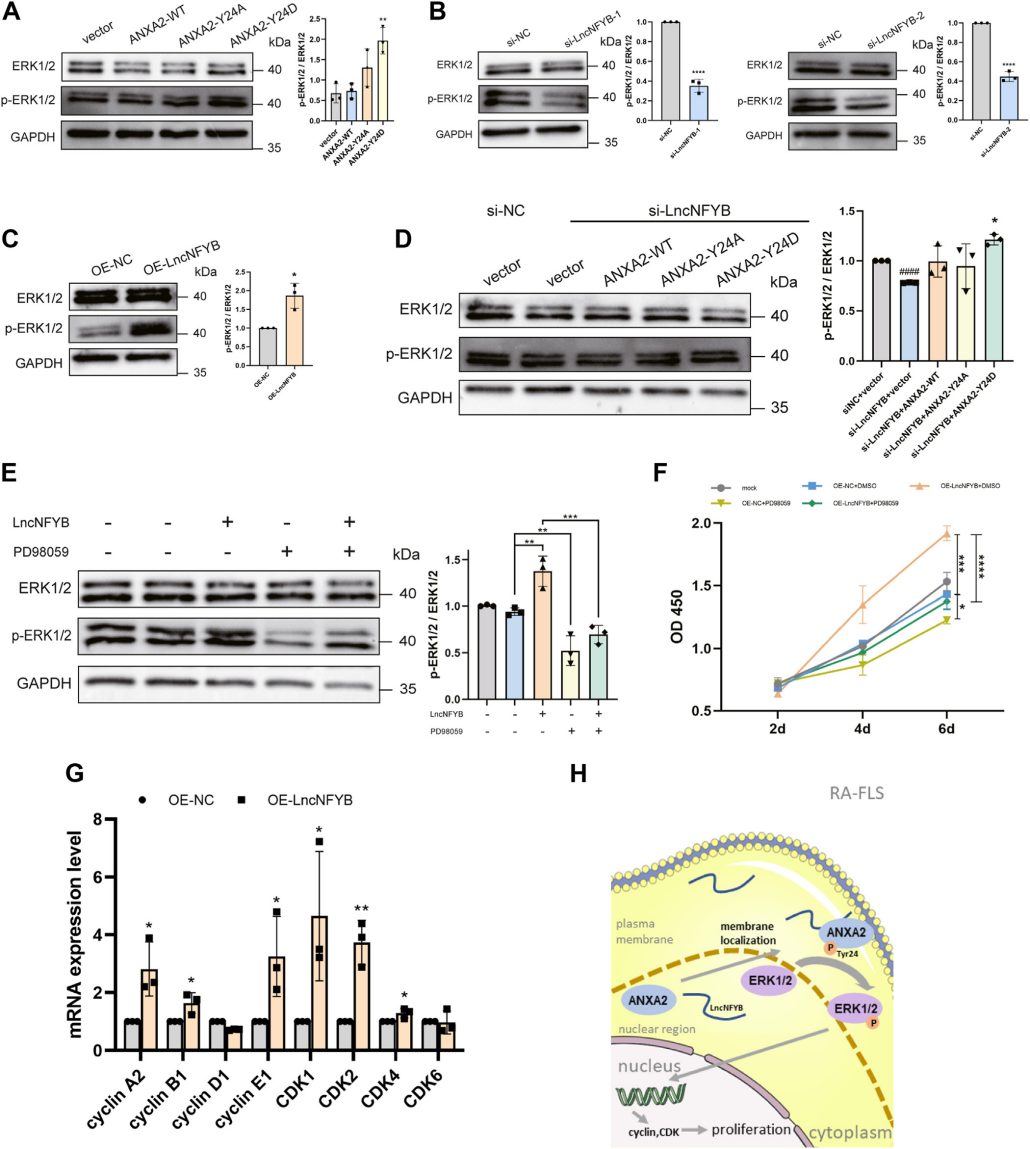
**LncNFYB promoted proliferation of RA-FLS *via* LncNFYB/ANXA/ERK1/2 axis.***A*–*D*, the expression levels of total proteins and phosphorylated proteins of ERK1/2 after transfection of mutant plasmids (*A*), LncNFYB knockdown (*B*), LncNFYB overexpression (*C*), and co-transfection of si-LncNFYB and ANXA2 plasmids (*D*). *E*, the expression levels of total proteins and phosphorylated proteins of ERK1/2 after LncNFYB overexpression and PD98059 co-treatment. The first column was the mock group, the second column was treated with control adenovirus and DMSO. PD98059 concentration: 50 μM. *F*, quantification of CCK-8 proliferation assays after LncNFYB overexpression and PD98059 treatment. *G*, the levels of expression of various cell cycle-related proteins after LncNFYB overexpression. *H*, schematic diagram of LncNFYB/ANXA2/ERK1/2 axis regulating cell proliferation in RA-FLS. "OE" indicates over-expression. ∗, ∗∗, ∗∗∗, and ∗∗∗∗ indicates to *p* < 0.05, 0.01, 0.001, and 0.0001 compared with the control group. *###* in (*D*) indicates *p* < 0.05 compared with the si-NC+vector group; ∗ in (*D*) indicates *p* < 0.05 compared with the si-LncNFYB +vector group. Data are represented as mean ± SEM (n = 3).

The ERK1/2 levels did not change in RA-FLS transfected with the plasmids, but the levels of p-ERK1/2 increased significantly in the ANXA2-Y24D overexpression group compared with that in the vector group (Fig. 8*A*). We also observed that the lack of LncNFYB resulted in the reduction of the p-ERK1/2 levels whereas LncNFYB abundance induced the phosphorylation of ERK1/2 (Fig. 8, *B* and *C*). A rescue experiment was performed to verify whether ANXA2 is an intermediary of LncNFYB to regulate ERK1/2. We co-transferred RA-FLS with siRNA and plasmids. Total ERK1/2 levels did not change, but the decrease in p-ERK1/2 caused by si-LncNFYB was rescued by ANXA2- Y24D transfection (Fig. 8*D*). Hence, we found that the LncNFYB/ANXA2/ERK1/2 axis exited in RA-FLS.

To further investigate whether ERK1/2 was downstream of LncNFYB to modify the proliferation, we blocked phosphorylation of ERK1/2 in LncNFYB overexpressed RA-FLS by inhibitor PD98059. PD98059 decreased the level of p-ERK1/2 of RA-FLS, and rescued the excessive p-ERK1/2 caused by LncNFYB (Fig. 8*E*). In CCK8 assay, the enhanced proliferation ability gained from exogenous LncNFYB was arrested by PD98059 (Fig. 8*F*). The result demonstrated that the proliferation regulation of the LncNFYB/ANXA2/ERK1/2 axis in RA-FLS.

Phosphorylation of ERK1/2 facilitates its nuclear translocation and promotes its activity as a transcription factor (21). Cyclin and CDK act downstream of ERK1/2 (22). We determined the mRNA levels of these proteins after LncNFYB overexpression. The transcription of cyclin A2, B1, E1, and CDK 1,2,4 was increased in LncNFYB-overexpressing RA-FLS (Fig. 8*G*).

In summary, this study identified that LncNFYB is overexpressed in RA-FLS and promotes its proliferation *via* the LncNFYB/ANXA2/ERK1/2 axis (Fig. 8*H*).

## Discussion

LncRNAs play important roles in the epigenetic regulation of various cellular activities. In this study, we identified LncNFYB, which is frequently upregulated in RA and promotes cell proliferation by promoting the phosphorylation of ANXA2, which further activates ERK1/2.

LncNFYB was highly expressed in RA-FLS, and promoted its proliferation. This proliferative effect also occurred in OA-FLS. Overexpression of LncNFYB allowed OA-FLS to develop a proliferation-enhancing disease phenotype like RA-FLS. These results indicated the increasing LncNFYB might be a cause of FLS over-proliferation in RA development.

To further investigate the mechanism of how LncNFYB affected proliferation, we did RNA pulldown then mass spectrometry. It was interesting that lncNFYB appeared to highly interacting with ribosomal proteins. This might be because of the following reasons: (1) It is combined with other structural domains of ribosomal proteins outside the mRNA recognized area. This combination might directly modify the mRNA translation. (2) LncNFYB could bind to RNA through base pairing, these binding-RNA might be mRNA or ribosomal RNA, which could interact with ribosomal proteins. These RNA might be as “bridge” for LncNFYB and ribosomal proteins. (3) We counted the number of a certain subset of pull-down proteins, and ribosomal proteins contain too many isoforms, rendering this subset contain the most richness.

ANXA2 is widely expressed in multiple cell types, including epithelial cells, stromal cells, mononuclear macrophages, and neurons (23, 24, 25, 26). It can be considered a tumor marker because it is often highly expressed in a variety of tumors, including lung, stomach, colorectal, liver, breast, cervical, and ovarian cancers (12, 27). ANXA2 can modify various pathways involved in pathophysiological processes, including tumor cell invasion, metastasis, bleeding disorders, angiogenesis, and induction of inflammatory factor expression (28, 29, 30, 31, 32, 33).

Only a few studies have been conducted on ANXA2 in patients with RA. Anti-ANXA2 antibodies have been detected in the serum of patients with RA and could provide a certain degree of diagnostic value for concomitant antiphospholipid syndrome (34). In patients with primary RA, ANXA2 expression levels were reported to be higher than normal and decreased significantly after treatment. Knockdown of ANXA2 in RA-FLS partially mimics the effects of glucocorticoids on cells, which alleviate the inflammatory level (35). In addition, ANXA2 can bind to CTGF in RA-FLS (36). and in vascular endothelial cells, it activates the downstream hedgehog (HH) signaling pathway (37), all of which promote pannus formation in RA.

The function of ANXA2 is determined by changes in its expression, post-transcriptional modifications (12), and binding partners. In RA-FLS, LncNFYB could bind to ANXA2 but did not change its expression; therefore, we wondered whether LncNFYB post-transcriptionally modified ANXA2. ANXA2 is expressed in both the cytoplasm and the nucleus of RA-FLS. Because LncNFYB was expressed only in the cytoplasm, it was more likely that LncNFYB affected ANXA2 located in the cytoplasm rather than in the nucleus.

Phosphorylation is a common post-transcriptional modification of ANXA2. The two most well-established phosphorylated sites are Ser25 (through the action of protein kinase C) and Tyr24 (through the action of SRC kinase), which was confirmed as functionally (15). Owing to the proximity of the two phosphorylation sites, phosphorylation at one site inhibits that at the other site; therefore, phosphorylation at the Ser25 and Tyr24 sites does not occur simultaneously (38, 39). The p-ANXA2 (Ser25) is primarily distributed in the perinuclear region, whereas p-ANXA2 (Tyr24) is predominantly distributed in the nucleus, cytoplasmic plasma membrane, and cytosol (40, 41, 42, 43). In tumors, and under oxidative stress, cytoplasmic Tyr24 phosphorylation promotes the translocation of ANXA2 to the cell membrane and promotes cell proliferation (20). We further put out the hypothesis that LncNFYB promoted phosphorylation of the Tyr24 site rather than Ser25.

Experiments show that LncNFYB promotes Tyr24 phosphorylation of its binding protein, ANXA2. Increased p-ANXA2(Tyr2) induced by hydrogen peroxide in fibroblasts and smooth muscle cells promotes ERK1/2 phosphorylation (20). In RA-FLS, the total amount of ERK1/2 protein was not associated with LncNFYB expression, but its activation was positively correlated. RA-FLS harboring the ANXA2-Y24D mutation plasmid had the highest p-ERK1/2 levels among the groups transfected with different plasmids. In rescue experiments, the ANXA2-Y24D group showed a reversal of the reduction in p-ERK1/2 caused by si-LncNFYB, suggesting the existence of the LncNFYB/ANXA2/ERK1/2 axis in RA-FLS.

ERK1/2 is a classical proliferation-regulating protein. p-ERK1/2 enters the nucleus, promotes the activation of nuclear kinases and regulates transcription factors, effectively promoting cell differentiation, proliferation, and cell cycle progression (44, 45). Cyclin and CDK family proteins are downstream of ERK1/2 and are directly involved in the cell cycle (46). Accordingly, LncNFYB improved the transcription of cyclin A2, B1, E1, and CDK 1, 2, and 4.

The activation of ERK1/2 enhances cell proliferation. It also affects cell migration and invasion (44, 45). However, LncNFYB was able to activate ERK1/2 and promote proliferation but had no effect on the migration and invasion abilities of RA-FLS. This might be due to cell type, frequency of ERK1/2 activation, and other regulatory mechanisms of LncNFYB.

It has been reported that H_2_O_2_ stimulation can promote the phosphorylation of vascular smooth muscle cells and fibrocytes ANXA2 on Tyr24, thereby promoting the activation of ERK1/2 (20). Combined with the microenvironment of RA-FLS in the affected joints, it is a chronic inflammatory environment with relatively high pressure and low oxygen, in which the content of oxygen free radicals increases and the cells are in a state of low-concentration oxidative stress. A low concentration of ROS can promote cell proliferation. Therefore, it is hypothesized that oxidative stress is one of the reasons for the increased expression of LncNFYB in RA-FLS, but specific experiments are needed to confirm this speculation.

In conclusion, we identified an increased lncRNA—LncNFYB—in RA-FLS, which promoted the proliferation and accelerated cell cycle progress of RA-FLS *via* the LncNFYB/ANXA2/ERK1/2 axis. Our findings revealed the pivotal role of LncNFYB in cell cycle control and hyperplasia in RA-FLS and implicated the LncNFYB/ANXA2/ERK1/2 axis as a potential therapeutic target for RA.

## Experimental procedures

### Isolation and culture of primary cells

Synovial tissue was obtained from patients with RA and osteoarthritis (OA). The patients with RA met the European League Against Rheumatism (EULAR) and American College of Rheumatology (ACR) 2010 RA classification criteria and those with OA met the 1995 ACR knee osteoarthritis classification criteria (47). The tissue samples were cut into small pieces that were allowed to adhere to culture flasks containing Dulbecco’s modified Eagle medium (DMEM; Gibco) supplemented with 10% fetal bovine serum (FBS) (Gibco). After FLS were extracted from the tissue, they were digested with trypsin (Gibco) to form a cell suspension, which was then subcultured into other bottles. The FLS were incubated in DMEM supplemented with 10% FBS at 37 °C in an atmosphere of 5% CO_2_. For experiments, fourth to tenth-generation FLS were used. The study was approved by the Ethics Committee of Southern Medical University (NFEC-20120201).

### Arraystar human lncRNA microarray

Total RNA was extracted from FLS obtained from three patients with RA and OA using TRIzol reagent (Invitrogen, USA). A high-throughput Arraystar human lncRNA microarray was used to determine the lncRNA expression levels in the RNA samples (Instrument: Agilent Microarray Scanner, Agilent p/n G2565BA). The lncRNA spectrum was retrieved from public databases (UCSC Known Genes, Ensemble, etc.). Bioinformatics tools were used to analyze the results. The information of microarray could be downloaded from GEO database (GSE243821).

### Quantitative real-time polymerase chain reaction (qRT-PCR)

Total RNA, extracted as described in the previous section, was reverse transcribed into cDNA using a PrimeScript RTase kit (Takara). qRT-PCR was performed on a LightCycler 480 (Roche) using TB Green Premix Ex Taq (Takara), according to the manufacturer’s protocol. Primers were designed using Primer Premier 6.0 and were synthesized by Tsingke Biotechnology Co.

### Fluorescence *in situ* hybridization (FISH) assay

Transferred the cells to cell crawling pieces, treated with 4% paraformaldehyde for 20 min, then methyl alcohol for 15 min at room temperature. The synthesis of LncNFYB probe and further processes were finished by Vipotion.

### siRNA transfection

The sequences of lncRNAs were retrieved from the UCSC Known Gene database (http://genome.ucsc.edu/). Specific siRNAs targeting LncNFYB were designed and synthesized by RiboBio. FLS (3 × 10^5^) were transferred to a 6-well plate before siRNA transfection. The stock siRNA solution (20 μM) was diluted into 50 nM working aliquots with a complete medium containing riboFECT CP reagent (RiboBio). After incubating at 37 °C in an atmosphere of 5% CO_2_ for 2 days, FLS were collected for further experiments.

### Overexpression of LncNFYB

FLS were transfected with an LncNFYB-overexpressing adenovirus for 24 h. After culturing for another 24 h, these FLS were used for further experiments. The multiplicity of infection (MOI) was 20.

### Western blotting

Premixed RIPA Lysis Buffer (Beyotime) was used to lyse FLS for 15 min after washing three times with precooled 1X PBS pH 7.4 (Gibco). The lysate was centrifuged at 14,000 rpm for 15 min at 4 °C. The supernatant, thus obtained, was boiled at 99 °C with 5 × DualColor protein loading buffer (FDbio) for 7 min and the mixture was subjected to sodium dodecyl sulfate-polyacrylamide gel electrophoresis. The voltage was set at 80 and 120 V during electrophoresis through the stacking and separating gels, respectively. The proteins were then electroblotted onto a 0.22 μm PVDF membrane (Millipore) at a constant current of 200 mA at 4 °C for 60 min. The membrane was blocked with a 5% bovine serum albumin solution at room temperature for 2 h, incubated overnight with primary antibody at 4 °C, and subsequently with secondary antibody at room temperature for 1 h. The blot was developed with FDbio-Pico ECL (FDbio) and the signal intensity was measured using a BLT GelView 6000 Pro Chemiluminescence gel imaging system (Biolight). The primary antibodies used were as follows: ANXA2 (1:1000; cat. no. ab235939; Abcam), p-ANXA2 (1:250; cat. no. sc-135753; Stanta Cruz), HNRNPC1/2 (1:1000; cat. no. ab133607; Abcam), NCL (1:1000; cat. no. ab129200; Abcam), p44/42 MAPK (ERK1/2) (1:5000; cat. no. ab184699; Abcam), p-p44/42 MAPK (ERK1/2) (1:1000; cat. no. ab50011; Abcam), and GAPDH (1:5000; Cat No. 60004-1-Ig; Proteintech), β-ACTIN (1:5000; Cat No. 81115-1-RR; Proteintech).

### CCK8 assay

A total of 8000 cells were transferred to a 96-well plate and experimentally treated for the required number of days. Thereafter, the culture medium was replaced with 100 μl/well of 10% cell counting kit-8 (CCK8) solution (Selleck) diluted with DMEM. The plate was kept at 37 °C for 3 h and the absorbance at 450 nm was read using a microplate reader. Exposure to light was avoided during the entire process.

### EdU assay

A total of 1.5 × 10^4^ cells were transferred to 48-well plates and incubated with culture medium containing 50 μM 5-ethynyl-2′-deoxyuridine (EdU) for 20 h. They were then observed under a fluorescence microscope (NIKON) and the total number of cells as well as the number of cells producing EdU fluorescence signal in each field of observation after staining with a Cell-Light EdU Apollo488 *In Vitro* Imaging Kit (RiboBio) was recorded.

### Cell cycle analysis

Cells (5 × 10^5^ cells) were collected in 75% alcohol at −20 °C overnight. The cell suspension was then mixed with propidium iodide (PI) solution in the Cell Cycle Analysis Kit (Multisciences) and incubated at room temperature for 30 min. Flow cytometry was used to determine the proportion of cells in different cell-cycle phases. The results were analyzed using the FlowJo V10 software.

### Apoptosis detection

Annexin V-APC/7-ADD apoptosis detection kit was used. Cells (5 × 10^5^ cells) were transferred into 500 μl 1 × working solution with 5 μl Annexin Ⅴ-APC solution and 10 μl 7-ADD solution. Incubated at room temperature and avoid light for 5 min before test. Software FlowJo used to analysis results.

### RNA pull-down assay

The LncNFYB probe was designed and synthesized by RiboBio. A Pierce magnetic RNA pull-down kit (Thermo Fisher Scientific) was used to obtain RBPs, according to the manufacturer's instructions. The protein solution was used for Western blotting and mass spectrometry.

### RNA immunoprecipitation (RIP)

Magnetic beads were conjugated with an antibody against a specific protein or with a homologous IgG antibody and then incubated with cell lysate, following the protocol provided with the Magna RIP RBP immunoprecipitation kit (12 reactions) (Merck). immunoprecipitated RNA associated with the RBP was purified and used for qRT-PCR or agarose gel electrophoresis.

### Plasmid transfer

The plasmids were designed and produced by Genecherm. Cells (5 × 10^5^) were transferred to 6-well plates and cultured in Opti-MEM (Gibco) containing the plasmid and Lipofectamine 3000 reagent (Invitrogen). After 24 h of incubation at 37 °C in an atmosphere of 5% CO_2_, the cells were collected and used for further experiments.

### Statistical analysis

Statistical significance was assessed using the Student’s *t* test (two-tailed) to compare two groups. For comparison among more than two groups, one-way analysis of variance (ANOVA) followed by Dunnett’s post-hoc test was used. A *p*-value <0.05 was considered statistically significant (∗*p* < 0.05, ∗∗*p* < 0.01, ∗∗∗*p* < 0.001 and, ∗∗∗∗*p* < 0.0001).

## Ethics approval

This study abided by the Declaration of Helsinki principles and was approved by the Institutional Ethics Committee of Southern Medical University, China (Approval Number; NFEC-20120201).

## Data availability

The datasets used or analyzed during the current study are available from the corresponding author on reasonable request.

## Supporting information

The article contains supporting information.

## Conflict of interest

The authors declare that they have no known competing financial interests or personal relationships that could have appeared to influence the work reported in this paper.

## Supporting information

Supplementary Figure *A*, the cluster diagram of the lncRNA microarray, the first three columns show lncRNA expression in RA-FLS from three patients, and the last three columns show lncRNA expression in OA-FLS from three patients. Green and red indicate relatively low and high expression levels, respectively. *B*, the expressions of ENST0000055540, ENST000005728, DQ570096., ENST00000576080, ENST00000550468, NR_144395, and ENST0000056319 in RA and OA FLS from five patients obtained from qRT-PCR. *C*, the level of LncNFYB after siRNAs knockdown. *D*, the level of LncNFYB after adenovirus overexpression. MOI = 20. *E*, the GO pathway of LncNFYB pulled-down proteins were analyzed. *F*, the protein-protein interaction network of proliferation-associated proteins from LncNFYB pulled-down were analyzed using the STRING database. *G*, the mRNA expression of ANXA2 after LncNFYB knockdown. *H*, the mRNA expression of ANXA2 after LncNFYB overexpression. *I*, mRNA expression of ANXA2 after transfection of the mutant plasmids. *J*, endogenous protein expression of exogenous ANXA2 fter transfection of the mutant plasmids. "OE" indicates over-expression. ∗, ∗∗, ∗∗∗, and ∗∗∗∗ indicates to *p* < 0.05, 0.01, 0.001, and 0.0001 compared with the control group. Data are represented as mean ± SEM (n = 3).
