# Supplementary figures and images for: LncNFYB promotes the proliferation of rheumatoid arthritis fibroblast-like synoviocytes via LncNFYB/ANXA2/ERK1/2 axis
Source: J Biol Chem. 2023 Dec 21;300(2):105591. doi: 10.1016/j.jbc.2023.105591 (PMC10867587; doi:10.1016/j.jbc.2023.105591)

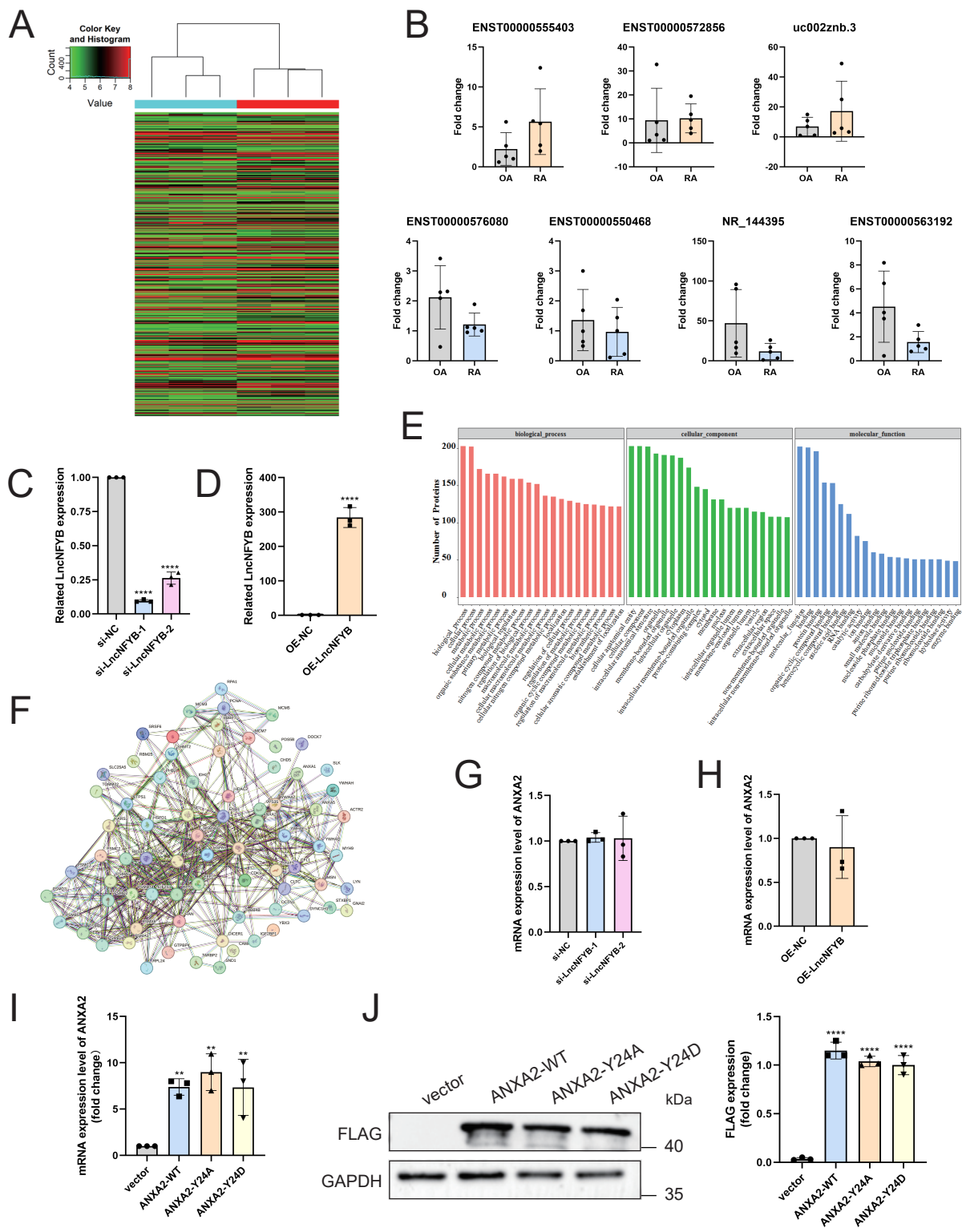

Supplement: Supplementary Figure — A, the cluster diagram of the lncRNA microarray, the first three columns show lncRNA expression in RA-FLS from three patients, and the last three columns show lncRNA expression in OA-FLS from three patients. Green and red indicate relatively low and high expression levels, respectively. B, the expressions of ENST0000055540, ENST000005728, DQ570096., ENST00000576080, ENST00000550468, NR_144395, and ENST0000056319 in RA and OA FLS from five patients obtained from qRT-PCR. C, the level of LncNFYB after siRNAs knockdown. D, the level of LncNFYB after adenovirus overexpression. MOI = 20. E, the GO pathway of LncNFYB pulled-down proteins were analyzed. F, the protein-protein interaction network of proliferation-associated proteins from LncNFYB pulled-down were analyzed using the STRING database. G, the mRNA expression of ANXA2 after LncNFYB knockdown. H, the mRNA expression of ANXA2 after LncNFYB overexpression. I, mRNA expression of ANXA2 after transfection of the mutant plasmids. J, endogenous protein expression of exogenous ANXA2 fter transfection of the mutant plasmids. "OE" indicates over-expression. ∗, ∗∗, ∗∗∗, and ∗∗∗∗ indicates to p < 0.05, 0.01, 0.001, and 0.0001 compared with the control group. Data are represented as mean ± SEM (n = 3). [file mmc1.pdf]
